# Efficacy of afoxolaner in a clinical field study in dogs naturally infested with *Sarcoptes scabiei*

**DOI:** 10.1051/parasite/2016026

**Published:** 2016-06-17

**Authors:** Frédéric Beugnet, Christa de Vos, Julian Liebenberg, Lénaïg Halos, Diane Larsen, Josephus Fourie

**Affiliations:** 1 Merial S.A.S. 29 avenue Tony Garnier 69630 Lyon France; 2 Clinvet International (Pty) Ltd PO Box 11186 9321 Universitas South Africa

**Keywords:** *Sarcoptes scabiei* var. *canis*, Sarcoptic mange, Afoxolaner, Efficacy

## Abstract

The acaricidal efficacy of afoxolaner (NexGard^®^, Merial) was evaluated against *Sarcoptes scabiei* var. *canis* in a field efficacy study, when administered orally at a minimum dose of 2.5 mg/kg to dogs naturally infested with the mites. Twenty mixed-breed dogs of either sex (6 males and 14 females), aged over 6 months and weighing 4–18 kg, were studied in this randomised controlled field efficacy trial. Dogs, naturally infested with *Sarcoptes scabiei* var. *canis* confirmed by skin scrapings collected prior to allocation, were randomly divided into two equal groups. Dogs in Group 1 were not treated. Dogs in Group 2 were treated on Days 0 and 28. On Days 0 (pre-treatment), 28 (pre-treatment) and 56, five skin scrapings of similar size were taken from different sites with lesions suggestive of sarcoptic mange. The extent of lesions was also recorded on Days 0, 28 and 56, and photographs were taken. Dogs treated orally with afoxolaner had significantly (*p* < 0.001) lower mite counts than untreated control animals at Days 28 and 56 with no mites recovered from treated dogs at these times (100% efficacy based on mite counts). In addition, dogs treated with NexGard had significantly (*p* < 0.05) better lesion resolution at Day 56 than Day 0; no treated dog showed pruritus compared to 7/10 dogs in the control group, 1/9 treated dogs had crusts compared to 5/10 controls and 8/9 dogs recovered 90% of hairs on lesions compared to 0/10 control dogs.

## Abstract (fr)

L’efficacité acaricide de l’afoxolaner (NexGard^®^, Merial), a été évaluée vis-à-vis de *Sarcoptes scabiei* var. *canis*, lors de l’administration par voie orale, à la dose minimale de 2.5 mg/kg, à des chiens naturellement infestés par les agents de gale au cours d’une étude de terrain. Vingt chiens de tout type et des deux sexes (6 mâles et 14 femelles), pesant 4 à 18 kg ont été inclus dans cette étude clinique de terrain, contrôlée et randomisée. Les chiens, confirmés comme naturellement infestés par *Sarcoptes scabiei* var. *canis* à l’aide de raclages cutanés avant leur allotement, étaient ensuite répartis de façon aléatoire dans deux groupes. Les chiens du groupe 1 n’étaient pas traités. Les chiens du groupe 2 ont été traités aux jours 0 et 28. Aux jours 0 (avant traitement), 28 (avant traitement), et 56, 5 raclages cutanés d’une même superficie ont été effectués sur 5 sites différents correspondant à des lésions de gale sarcoptique sur chaque chien. L’extension des lésions a aussi été enregistrée aux jours 0, 28, et 56, et des photographies ont été prises. Les chiens traités oralement avec l’afoxolaner ont présenté significativement (*p* < 0.001) moins d’acariens que les chiens non traités aux jours 28 et 56, puisqu’aucun sarcopte n’a été retrouvé sur ces chiens lors de ces jours d’observation (soit une efficacité de 100 % sur la base des comptages d’acariens). De plus, les chiens traités au NexGard ont présenté des scores cliniques significativement inférieurs (*p* < 0.05) au jour 56 par rapport à J0 ; aucun des 9 chiens traités ne présentait de prurit contre 7/10 chiens contrôles, un chien traité avait encore des croûtes contre 5/10 contrôles, et 8/9 chiens traités présentaient une repousse du poil supérieure à 90 % de la surface des lésions contre 0/10 chien contrôle.

## Introduction

Canine sarcoptic mange is a highly contagious, intensely pruritic parasitic skin disease caused by infestation with the epidermal mite *Sarcoptes scabiei* var. *canis* (DeGeer, 1778) [16, 19]. The disease commonly affects domestic dogs, especially young animals, with a worldwide distribution. It is a highly contagious and zoonotic mite; half of the dogs and up to 50% of their human companions can develop skin lesions after having contact with infested dogs [20]. Fortunately, the mites cannot breed in human skin and humans are a dead-end host for the canine variety, usually showing a transitory erythematous and papular skin reaction on some part of their body only. Additionally, *S. scabiei* var. *canis* has been isolated from species other than domestic and wild canids and has been experimentally established on several mammals of other species. This lack of host specificity has therapeutic and public health implications [16, 19, 20]. Diagnosis in dogs is based on the presence of clinical signs (infested dogs show severe pruritus, an erythematous rash and yellowish crusts on the skin), the detection of mites on skin scrapings and/or the response to acaricidal therapy. Under practical veterinary conditions, *Sarcoptes* mites are difficult to find, and several skin scrapings are necessary to observe a few mites [19]. In efficacy studies including dogs showing severe skin lesions, finding mites by performing at least five skin scrapings of 4 cm^2^ is not too difficult [13, 17, 21, 23, 25].

There are various topical and systemic therapies currently used for the treatment of sarcoptic mange in dogs [4, 7], either by contact activity onto the skin (e.g. amitraz dips; fipronil spray, metaflumizone/amitraz spot-on formulation, imidacloprid/flumethrin collar; or fipronil/amitraz/S-methoprene spot-on formulation) [6, 8, 14, 15, 16, 19, 20, 24], or through systemic activity (e.g. selamectin and moxidectin/imidacloprid spot-on formulation having transcutaneous absorption) [13, 17, 21, 23, 25]. Although they are reported as being generally effective treatments for sarcoptic mange in dogs, some have limitations associated with their topical application. Further, shampooing or bathing with medicated or non-medicated formulations are part of the adjunctive therapy for clinical mange in order to rehydrate the skin and treat seborrhoea, but these procedures may reduce the efficacy or shorten the sustained activity of topical products [7, 19]. Two oral products are approved in some countries for the treatment of sarcoptic mange. The first is milbemycin oxime, however the need for administration every two days may not be a convenient option for dog owners [7, 19]. The second is sarolaner (Simparica^TM^, Zoetis), belonging to the antiparasitic isoxazolines [1]. Afoxolaner is a novel molecule belonging to the antiparasitic isoxazolines, which is used orally at the minimum effective dose of 2.5 mg/kg to control fleas and ticks in dogs (NexGard^®^, Merial) [9, 11, 18, 22]. Afoxolaner provides a curative effect on existing fleas and ticks and a sustained preventive effect for a month against new flea or tick infestations. A recent clinical study demonstrated its efficacy against canine generalised demodicosis, one of the most challenging ectoparasitoses to treat in dogs [5]. Efficacy of afoxolaner has been demonstrated against *Sarcoptes scabiei* var. *suis* in a pig model using NexGard^®^ at a dose of 2.5 mg/kg [2, 3]. The present study was designed to evaluate the efficacy of afoxolaner against natural infestation with *Sarcoptes scabiei* var. *canis*, when administered to dogs at the commercial dose.

## Materials and methods

### Study design

This study was a parallel-group, randomised, controlled field efficacy study [10]. The study was conducted on two groups of 10 dogs each. Start of the study was on different days for the dogs, as dictated by logistical constraints. Ten dogs remained untreated in Group 1 which served as a control and 10 dogs in Group 2 were treated orally with afoxolaner (NexGard^®^) at a dose of at least 2.5 mg/kg based on their weight (Table 1).

**Table 1.** Tabulated study design.

Allocation into groups	Administration of NexGard^®^	Mite counts and clinical symptom assessments (including photographic documentation)	Body weights	Clinical examinations
Day −1/0	Days 0 and 28	Days −1/0, 28 and 56	Days −1/0, 28 and 56	Days −1/0, 28 and 56

The experimental unit was the individual dog. Due to the unavailability of a reliable model of *Sarcoptes scabiei* infestation, dogs included in this clinical trial were privately owned dogs. The study design was approved by the ClinVet Committee for Animal Ethics and Welfare (CCAEW), in accordance with the South African National Standard on the care and use of animals for scientific purposes (SANS 10386:2008 – Edition 1) Annex F, F.1.2. An Informed Consent and Agreement form was completed and signed by each owner before any study-related activities began. The animals were handled in compliance with Merial Institutional Animal Care and Use Committee (IACUC) approvals and with due regard for their welfare. It was agreed with the owners that the untreated dogs would receive a curative approved treatment on Day 56 (Advocate^®^, Bayer) and that they would undergo rescue treatment at any time if their clinical status worsened or if this was requested by their owner.

Randomisation of animals to treatment groups, as well as administration of the treatment, was the responsibility of a non-blinded member of the study personnel. After randomisation, non-blinded personnel were not involved in any other experimental procedures. All other people involved in the study were blinded to the group allocation.

All dogs were aged > 6 months, including 6 males and 14 females weighing 4–18.3 kg. On Day 0, the arithmetic mean body weight was 10.4 kg (range 4 kg–14.5 kg) for the negative control group, and 10.9 kg (range 4.2 kg–18.3 kg) for the afoxolaner-treated group. The body weights did not differ significantly between the groups (*p* = 0.7987). The dogs were healthy at the initiation of the study on Day −1/0, with the exception of clinical signs associated with sarcoptic mange. To be included in the study, they had to harbour live *S. scabiei* mites, as diagnosed by skin scrapings. Presence of *Demodex* spp mites, treatment with glucocorticoids, or any ectoparasiticide or macrocyclic lactone in the 12 weeks prior to Day 0 were criteria for exclusion.

The dogs stayed with their owners under their usual housing conditions for the duration of the study (Table 1). Food and water were offered according to the usual habits of the owners.

The dogs from Group 2 were treated orally with afoxolaner (NexGard^®^, Merial), based on their weight, on Days 0 and 28, following the commercial registration labelling [11].

### Specification of study variables

#### Mite counts

The efficacy evaluation was primarily based on the reduction of *Sarcoptes scabiei* mites counted in skin scrapings, and treatment efficacy was presented as the percentage reduction of mites per treatment group as well as the number of mite-free dogs compared to the controls.

Skin scrapings (±4 cm^2^) were performed on Days −1/0, 28 and 56 from five different body areas suspected of being infested due to the observation of lesions (crusts, hair loss, erythema and/or papules). The skin scraping sites were recorded for each dog and re-used for the next scrapings. Each scraping material was transferred to a marked (animal ID and body region) microscope slide containing mineral oil and was examined under a stereomicroscope for the presence of live mites. The number of mites counted in each scraping was recorded.

#### Lesion score

Pruritus was assessed on each dog on the days when scrapings were made and recorded on a standardised form. Its presence or absence was documented during a 5-minute observation period. The number of dogs showing pruritus in treated versus control dogs was compared (Table 3).

Similarly, the presence of crusts on any part of the dog’s body was assessed and the number of animals presenting crusts in treated versus control dogs was compared (Table 3).

Crusts and hair loss were sketched on a dog silhouette (left- and right-hand sides): body areas covered by crusts; body areas with hair loss (1 = slight thinning of hair; 2 = conspicuous hair loss; 3 = no hair).

Coloured photographs to illustrate the extent of lesions and their resolution were taken of each dog before treatment (Day −1/0) and on Days 28 and 56 (Photographic documentation).

### Data analysis

#### First criterion = reduction in mite counts

The difference in the number of mites between treated and control animals was calculated. A percentage reduction in mite counts > 95% was considered to be the first criterion for treatment success. Efficacy was calculated for the treated dogs at each assessment Day according to the formulas given below:

Percentage reduction=100×(Mc-Mt)/Mc,

where:

Mc = Mean number of mites on all dogs in the control group (group 1) at a specific time point.

Mt = Mean number of mites on all dogs in the NexGard^®^ group (group 2).

#### Second criterion = mite-free dogs based on mite counts

The absence of mites on a dog was considered as the second criterion for assessment of treatment success. The proportion of the total number of dogs in each of the two groups (mite-free dogs) was calculated by:

$$\mathrm{Mite}-\mathrm{free}\;\mathrm{dogs}\;(\mathrm{MFD})=\frac{\mathrm{Number}\;\mathrm{of}\;\mathrm{dogs}\;\mathrm{with}\;\mathrm{an}\;\mathrm{absence}\;\mathrm{of}\;\mathrm{mite}\;\mathrm{counts}}{total\;\mathrm{nu}m\mathrm{ber}\;\mathrm{of}\;\mathrm{dogs}}$$

Failure rate (FR) = 1 – MFD.

The complete efficacy was then calculated as follows:

Cured dogs (%)=(1-FRT/FRC)×100%,

where:

FRT = the failure rate of the treated group and FRC = the failure rate of the control group.

### Resolution of clinical signs and symptoms

The presence or absence of pruritus and crusts on a dog, and the extent of alopecia, were used to determine clinical response. The number of dogs without either pruritus or crusts was determined at each assessment on Days 0, 28 and 56. The clinical efficacy was evaluated based on the resolution of the clinical sign from baseline for pruritus or crusts and calculated as [(observation of the sign at Day 0 − observation of the sign at Day 28 or 56)/observation of the sign at Day 0] × 100. Furthermore, semi-quantitative assessment of hair re-growth was also done compared to Day 0 baseline, and a score awarded to each dog on Day 56 (Tables 3 and 4).

### Statistical methods

Due to the risk of self-cure in the control group, a sample size of 10 dogs per group was used to increase the validity of the results.

Due to the fact that small and even zero mite counts could be recorded, it can be expected that the mite counts did not follow a normal distribution. Therefore, statistical analysis was based on a non-parametric Mann-Whitney U-test.

Based on EMA/CVMP efficacy guidelines and the threshold for fleas, the treatment was considered effective if at least a 95% reduction in mite counts was recorded on the final assessment day (Day 56).

In regard to crusts, pruritus and hair re-growth, the proportions of dogs in the absence/presence of categories, or % hair re-growth, were compared with a Fisher’s exact test between Day 0 and Day 56.

## Results

At the start of the trial, all dogs presented with skin abnormalities and clinical signs consistent with sarcoptic mange infestations, which included alopecia, papules, dermatitis, erythema and/or crusts. One treated dog (869 E65) died during the study and was not seen by the veterinarian for its second visit, the cause of death was accidental and not related to the treatment.

Nine of the ten animals in the negative control group retained their mite infestation up to Day 56 (±2 days), indicating viable mite infestation during the study. The arithmetic mean number of mites recorded for the dogs in the negative control group ranged from 46.8 to 92.8 (Table 2).

**Table 2.** Results of mite counts by study day.

		Day 0	Day 28	Day 56
Animal ID	Group	Number of mites	Number of mites	Number of mites
	Group 1 – Untreated			

885 FB9		59	13	0
869 C14		22	4	4
698 08C		61	14	15
86C 2A1		6	16	12
885 EA0		127	141	743
86A E35		10	5	16
1E0 190		8	1	1
886 0A1		331	268	15
86A 9D2		14	0	2
86A 075		11	6	120
	Average mite count (arithmetic mean)	64.9	46.8	92.8

	Group 2 – Afoxolaner treated			

86A E52		54	0	0
86A E8D		9	0	0
697 F75		45	0	0
86F C17		26	0	0
86A DD4		11	0	0
698 20F		26	0	0
86A AC2		649	0	0
86A C6B		4	0	0
86A DE4		14	0	0
869 E65		331	–	–
	Average mite count (arithmetic mean)	116.9	0.0	0.0
	% Efficacy		100%	100%
	*p*-value (Mann-Whitney U-test)	Not significant	0.00108	0.00108

For the NexGard^®^-treated group, the arithmetic mean number of mites was reduced from 116.9 (on Day 0) to 0 on both Day 28 (±2 days) and Day 56 (±2 days). Statistically significant (*p* < 0.05) fewer mites were recorded for the treated group compared to the untreated control group on Days 28 (±2 days) and 56 (±2 days). The treatment was 100% effective against *Sarcoptes scabiei* infestations on both Days 28 (±2 days) and 56 (±2 days).

The second criterion was the number of mite-free dogs. NexGard^®^ was successful in eliminating *Sarcoptes scabiei* mites from 9/9 treated dogs (100%) in comparison with the negative control, where only 1/10 dogs naturally cleared infestation and was mite-free on Days 28 (±2 days) and 56 (±2 days).

The third assessment criterion involved evaluation of clinical signs (Table 3). Resolution of clinical signs was compared against baseline (Day 0) for both Day 28 (±2 days) and Day 56 (±2 days). A significant change in the proportion of dogs showing signs to dogs without signs was recorded for pruritus on Day 28 (±2 days) and pruritus together with crusts on Day 56 (±2 days) (Chi-square test, *p* < 0.05). Control dogs (7/10) presented crusts on Day 0, 7/10 on Day 28 and 5/10 on Day 56, compared to 5/9, 2/9 and 1/9 treated dogs, respectively. Control dogs (4/10) showed pruritus on Day 0, 8/10 on Day 28 and 7/10 on Day 56, compared to 9/9, 0/9 and 0/9 treated dogs, respectively.

**Table 3.** Resolution of the clinical signs related to *Sarcoptes scabiei* var. *canis.*

		Frequency of sign		Efficacy (= resolution of the clinical sign from baseline) (%)
Day	Sign	Group 1	Group 2	Group 2
Day 0	Pruritus	4/10	9/9	/
Day 0	Crusts	7/10	5/9	/
Day 28	Pruritus	8/10	0/9	100%
Day 28	Crusts	7/10	2/9	60%
Day 56	Pruritus	7/10	0/9	100%
Day 56	Crusts	5/10	1/9	80%

Group 1: Negative control.

Group 2: Dogs treated orally with NexGard^®^.

All animals enrolled in the study had slight/conspicuous hair loss or patches with no hair (Table 4). The percentage of hair re-growth was significantly greater for treated dogs on Day 56 compared to baseline, and hair re-growth was also significantly better for treated versus control dogs at this time with 8/9 dogs showing hair re-growth >90%, compared to 0/10 control dogs (Chi-square test, *p* < 0.05).

**Table 4.** Estimated percentage of hair re-growth from baseline and between groups.

Day	Estimated percentage of hair re-growth					
	Group 1 (number of dogs/number of dogs per group)			Group 2 (number of dogs/number of dogs per group)		
	0–50%	50–90%	>90%	0–50%	50–90%	>90%
0	10	0	0	9	0	0
56 (±2 days)	6	4	0	0	1	8
Score	Description					
1	Body areas with hair re-growth 0%–50% compared to that recorded during the pre-treatment assessment					
2	Body areas with hair re-growth > 50% to < 90% compared to that recorded during the pre-treatment assessment					
3	Body areas with hair re-growth ≥ 90% compared to that recorded during the pre-treatment assessment					

Group 1: Negative control.

Group 2: Dogs treated orally with the IVP NexGard^®^.

Due to the efficacy of NexGard^®^ in this study, this treatment was offered to the owners of the dogs in the control group on Day 56 instead of Advocate^®^. Five dogs could be evaluated 28 days later. The arithmetic mean number of mites was reduced from 30.8 (on Day 56 ± 2 days) to 0.2 on Day 84 (±2 days) (Table 5). In only 1/5 dogs, a single mite was observed during the Day 84 (±2 days) assessment.

**Table 5.** Efficacy of treatment on five control dogs at Day 56 (based on arithmetic means).

Five control dogs from Group 1	Arithmetic mean of *Sarcoptes* mites		*p*-value
Days	Day 56 (±2 days)	Day 84 (±2 days)	
Mean (Eff%)	30.8	0.2 (99.4%)	*p* < 0.01

*p*-value: Mann-Whitney U-test.

Group 1: Dogs received NexGard^®^ on Day 56 (±2 days).

## Discussion

The 20 animals evaluated in this study were representative of the target dog population since there are no reported breed, sex or age predilections for sarcoptic mange [12, 19].

In this study, a single treatment given orally with afoxolaner resulted in rapid and complete cure of *Sarcoptes scabiei* infestation in 1 month and in the resolution of clinical signs in most dogs in 1–2 months. No adverse events were observed following the treatment administrations. The number of mites decreased from an average of 166.9 to 0 in the treated group at both Day 28 and Day 56. A few adult mites or eggs may have been missed due to the sensitivity of the skin scraping technique, especially at Day 28, but the observed results at Day 56 and the clinical improvement support the complete cure of the dogs. During the treatment of the control dogs with afoxolaner at the end of the study, one single mite was found among the five “control” dogs treated. This is consistent with the fact that a few mites may still be present after a month, indicating that a second treatment is probably needed to reach complete antiparasitic efficacy in any case.

The results of this study show complete parasitological cure of sarcoptic mange in dogs following monthly treatment with an oral ectoparasiticide under field conditions. This is consistent with findings reported for another isoxazoline molecule, sarolaner [1]. In other publications, elimination of mites in skin scrapings has been reported for topical products including selamectin [21], imidacloprid/moxidectin [13] and amitraz/fipronil/S-methoprene [15] after two monthly administrations in laboratory studies. In dogs fitted with an imidacloprid/flumethrin collar, the parasitological cure was reported after 3 months in a laboratory study [24]. In several studies, either no control group was included [14, 24] or a positive control product was used [13]. In the laboratory studies that used placebo-treated [21] or untreated control animals [15], a decrease in mite counts was reported in the control animals during the study period. In a recently reported sarolaner laboratory study, no mites were found in the skin scrapings from 9 out of the 11 untreated dogs that did not receive immunosuppression [1]. These observations suggest that mite counts in laboratory studies could be impacted by spontaneous cure. In the field study reported here, only 1 out of the 10 control dogs became negative for mites in skin scrapings, but the others remained positive, even with increasing mite counts, and no improvement of their skin conditions as visible on the photographic documentation. The observed self-cure could be related to an improvement of the immunological status and response of this particular animal. It could also be related to a change in food, in mode of life, or in age [16, 19], but it was not further investigated. Randomised, controlled multi-centre field studies have been reported for selamectin [23] and for imidacloprid/moxidectin [17]. In both of these studies, parasitological cure was achieved following two monthly applications and this was accompanied by an improvement in pruritus and skin lesions characteristic of sarcoptic mange. In the field study assessing sarolaner efficacy, the parasitological efficacy rates were 88.7% and 100% in the sarolaner group and 84.6% and 96.0% in the imidacloprid/moxidectin group, on Days 30 and 60, respectively, and pruritus resolved in all but one sarolaner-treated dog [1]. In the afoxolaner field study reported here, complete parasitological cure was observed within the first month and pruritus, the most obvious clinical sign of sarcoptic mange, also resolved in all dogs within a month. Sarcoptic mange may occur in dogs of all ages but its prevalence is higher in young dogs [12], and afoxolaner, because of its safety profile, can be used in dogs as early as 8 weeks of age [9, 11].

## Conflict of interest

This clinical study was funded by Merial S.A.S., 29 avenue Tony Garnier, 69007 Lyon of which Frédéric Beugnet, Lénaïg Halos and Diane Larsen are employees.

ClinVet, of which Christa de Vos, Julian Liebenberg and Josephus Fourie are employees, is an independent South African Contract Research Organisation contracted to conduct the study in the field.

All authors voluntarily publish this article and have no personal interest in these studies other than publishing the scientific findings that they have been involved in via planning, initiating, monitoring and conducting the investigations and analysing the results.

## Disclaimer

NEXGARD^®^ is a registered trademark of Merial. All other brands are the property of their respective owners.

This document is provided for scientific purposes only. Any reference to a brand or a trademark herein is for informational purposes only and is not intended for a commercial purpose or to dilute the rights of the respective owner(s) of the brand(s) or trademark(s).
